# High-Sensitivity and Wide-Range Flexible Pressure Sensor Based on Gradient-Wrinkle Structures and AgNW-Coated PDMS

**DOI:** 10.3390/mi16040468

**Published:** 2025-04-15

**Authors:** Xiaoran Liu, Xinyi Wang, Tao Xue, Yingying Zhao, Qiang Zou

**Affiliations:** 1 School of Microelectronics, Tianjin University, Tianjin 300072, China; 2022232072@tju.edu.cn; 2 Tianjin Flying Pigeon Group Co., Ltd., Tianjin 301600, China; 13116091576@163.com (X.W.); 18622912991@163.com (Y.Z.); 3 Center of Analysis and Testing Facilities, Tianjin University, Tianjin 300072, China; xuetao@tju.edu.cn; 4 Tianjin International Joint Research Center for Internet of Things, Tianjin 300072, China; 5 Tianjin Key Laboratory of Imaging and Sensing Microelectronic Technology, Tianjin University, Tianjin 300072, China; 6 State Key Laboratory of Advanced Materials for Intelligent Sensing, Tianjin University, Tianjin 300072, China

**Keywords:** flexible pressure sensors, gradient-wrinkle structure, silver nanowire, piezoresistive sensor, human motion monitoring

## Abstract

Flexible pressure sensors have garnered significant attention due to their wide range of applications in human motion monitoring and smart wearable devices. However, the fabrication of pressure sensors that offer both high sensitivity and a wide detection range remains a challenging task. In this paper, we propose an AgNW-coated PDMS flexible piezoresistive sensor based on a gradient-wrinkle structure. By modifying the microstructure of PDMS, the sensor demonstrates varying sensitivities and pressure responses across different pressure ranges. The wrinkle microstructure contributes to high sensitivity (0.947 kPa^−1^) at low pressures, while the PDMS film with a gradient contact height ensures a continuous change in the contact area through the gradual activation of the contact wrinkles, resulting in a wide detection range (10–50 kPa). This paper also investigates the contact state of gradient-wrinkle films under different pressures to further elaborate on the sensor’s sensing mechanism. The sensor’s excellent performance in real-time response to touch behavior, joint motion, swallowing behavior recognition, and grasping behavior detection highlights its broad application prospects in human–computer interaction, human motion monitoring, and intelligent robotics.

## 1. Introduction

With the rapid advancement in flexible electronics, flexible sensors have demonstrated significant potential for applications in health monitoring [1,2], smart wearable devices [3], and robotic haptic feedback [4]. Among pressure sensors based on various sensing mechanisms, such as piezoresistive [5], capacitive [6], and piezoelectric [7], piezoresistive pressure sensors are widely used in flexible pressure sensors due to their simple structure and excellent sensitivity [8,9,10]. In recent years, improving the sensitivity of piezoresistive sensors has been a major focus of research [11,12]. Traditionally, methods to enhance sensitivity include the introduction of microstructures and increases in material surface area. In particular, incorporating microstructures such as domes [13] and micro-pyramids [14] on the surface of elastomers can significantly increase the contact area, thereby improving sensitivity. For instance, Cui et al. [15] designed a synergistically microstructured flexible pressure sensor with a fiber and microdome (FMD) structure, consisting of a fiber conductive layer sandwiched between the PDMS microdome layer and an interdigital electrode. Due to the synergistic effect of multiple deformations within the FMD structure, the sensor resistance changes sensitively and continuously under pressure, resulting in a high sensitivity of 6.31 kPa^−1^. However, the fabrication process of these microstructures is complex and costly [16,17], presenting significant challenges for equipment and large-scale manufacturing. The surfaces of many organisms in nature are rich in microstructures that enable excellent external pressure sensing capabilities [18,19]. For example, He et al. [20] proposed a flexible pressure sensor based on a 3D interlocking structure inspired by the biomimetic dog-tail grass. The TPU and zinc oxide nanowires in this structure are interlocked, and, when pressure is applied, the contact area expands proportionally with the increase in pressure, achieving a high sensitivity of 29.7 kPa^−1^. Although biomimetic structures exhibit good performance, the difficulty in ensuring regularity and controllability during the fabrication process often leads to unstable sensor performance and increased fabrication complexity.

In contrast, wrinkle patterns have become a simple and low-cost solution that is gradually emerging as an effective method to enhance sensor performance. The wrinkle structure not only improves sensitivity by increasing the contact area but has also become a hot research topic in recent years due to its controllability and low cost [21,22,23]. For example, Lv et al. [24] proposed a spontaneously wrinkled MWCNT/PDMS dielectric layer to achieve excellent sensitivity and linearity in capacitive sensors for tactile sensing. In this context, the generation of wrinkle structures on polydimethylsiloxane (PDMS) surfaces using ultraviolet ozone (UVO) irradiation has become a common strategy [25,26]. UVO irradiation leads to the oxidation of the PDMS surface, which alters the surface tension and, consequently, facilitates the formation of wrinkles [27]. For example, Cho et al. [28] used opaque glass and stretched polydimethylsiloxane templates to fabricate novel sensing materials with convex and randomly folded microstructures in a cost-effective and straightforward manner, thereby preparing pressure sensors with high sensitivity. To further enhance sensor performance, recent studies have explored the generation of patterned wrinkle structures on the PDMS surface using mask technology. This method allows for precise control over the shape and distribution of the wrinkles at the micrometer scale, thereby improving sensor performance [29,30]. Although sensors based on wrinkle structures generally exhibit high sensitivity, their limited measurement range restricts their applicability in certain fields. Therefore, extending the measurement range of these sensors has become an urgent challenge. To address this, some researchers have proposed the incorporation of a gradient-wrinkle structure to expand the measurement range [31,32]. For example, Lv et al. [33] fabricated graphene-based pressure sensors by combining pre-stretching and laser reduction strategies to create a gradient-wrinkle structure in the graphene oxide layer, which endows the sensor with a wide detection range. Conventional gradient-wrinkle structures are typically achieved by controlling the film thickness; however, this method often results in irregular wrinkles, which in turn affect the sensor’s performance [34,35].

To address this issue, a new method is proposed in this paper for preparing AgNW-coated PDMS flexible piezoresistive sensors with gradient wrinkles. This method involves accurately controlling the mask position and UV irradiation time while using a stepper motor to pull the mask at a uniform speed. The wrinkle microstructure provides the sensor with a large contact area, resulting in high sensitivity, while the gradient-height wrinkle design ensures a continuous increase in the contact area through the gradual activation of the wrinkles, contributing to a wide sensing range. Silver nanowires (AgNWs) are an excellent choice for the conductive layer due to their high conductivity and mechanical flexibility. When spray-coated onto PDMS, they offer low cost and high efficiency. Additionally, the random AgNW network, with its high aspect ratio, is less prone to breakage under stretching. Notably, the entire fabrication process does not require expensive or complex techniques, and the wrinkle morphology and sensor sensitivity are effectively enhanced through UV irradiation treatment, motor speed control, applied pre-strain, and optimization of the PDMS thickness. The optimized sensor exhibits a high sensitivity of 0.947 kPa^−1^ and a lower detectable pressure limit of 10 Pa. In the load–unload test, the sensor demonstrated excellent stability over 3000 cycles, a fast response time of 80 ms, and a quick recovery time of 115 ms. Touch behavior, different frequencies of pressing tests, and finger and elbow joint movements highlight the sensor’s real-time detection capabilities in human–computer interaction and human motion monitoring. Additionally, the detection of swallowing behavior and water cup grasping experiments further demonstrate the sensor’s promising application in intelligent robotic systems.

## 2. Experimental Details

### 2.1. Fabrication Process of Gradient-Wrinkled AgNW-Coated PDMS Piezoresistive Sensors

**Figure 1:**
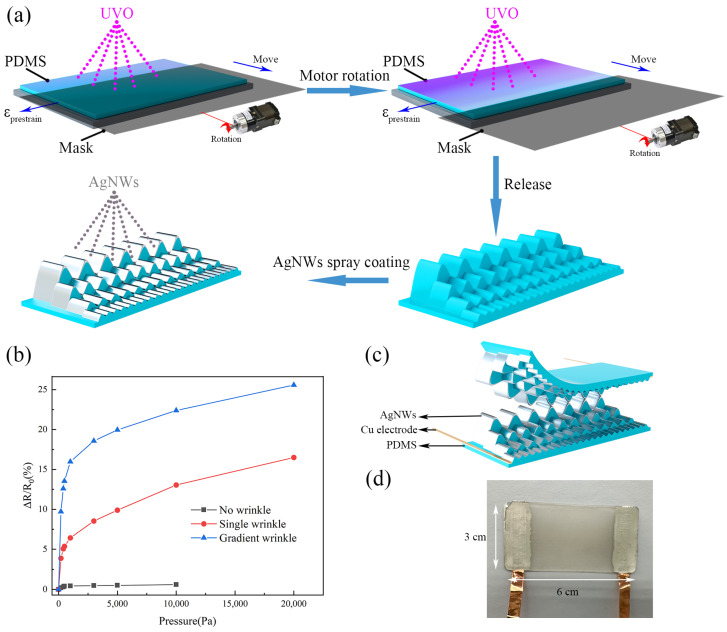
(**a**) Process flow for the preparation of AgNW-coated PDMS layers with gradient-wrinkle surfaces. (**b**) Relative resistance variation of the three sensors. (**c**) Device structure diagram of the gradient-wrinkle sensor. (**d**) Photographs of the sensors.

Figure 1a shows a schematic diagram of the preparation process of PDMS coated with AgNWs on gradient-wrinkle surfaces. PDMS was purchased from Dow Corning, and AgNWs were purchased from Shandong Lite Nanotechnology Co., Jining, China. First, the PDMS films were uniaxially pre-stretched using a stretching device, followed by 40 min of UVO treatment. To adjust the wavelengths of the wrinkles in different regions, we control the position of the mask using stepper motors, allowing for precise regulation of the UV exposure time in each region and control of the ozone concentration. After releasing the pre-stretching, a wave-like wrinkle structure is formed on the surface of the film due to the difference between the surface and internal Young’s moduli of PDMS. At the same time, the variation in light exposure time leads to changes in the thickness of the oxide layer, in turn forming gradient wrinkles with different heights. Next, AgNWs are sprayed onto the wrinkle surface to obtain an AgNW-coated PDMS film with gradient wrinkles. Finally, the two films are assembled face-to-face to construct the sensor, with copper foil used as the electrode, as shown in Figure 1c. To ensure that the initial resistance of the sensor remains constant and to prevent tilting of the upper PDMS film, we used double-sided adhesive as a support layer between the two wrinkle films. This structure ensures that the pressure applied to different areas of the sensor is evenly distributed, with the highest wrinkles being contacted first. For comparison, we fabricated flexible piezoresistive sensors with different film structures using PDMS films coated with AgNWs, including sensors with no wrinkles, single wrinkles, and gradient wrinkles, as shown in Figure 1b. Additionally, we fabricated the sensors under various experimental conditions, including different UVO processing times, stepper-motor-controlled mask speeds, applied pre-strain, and PDMS film thickness. Figure 1d shows a photograph of the final sensor.

### 2.2. Characterization

The sensor resistance was measured using an LCR meter (IM3523, HIOKI, Tokyo, Japan). Specific pressure was applied using a force gauge (AIGU-ZP-100, ETOOL Co., Ltd., Tokyo, Japan) combined with a self-assembled high-precision displacement stage (42BYG4812AA, Huisitong Co., Ltd. Wuxi, China). The test system consists of a computer, an LCR meter, and a pressure system. The pressure system applies pressure to the transducer in the vertical direction, while the LCR meter and computer software are responsible for measuring and recording data, respectively. For cyclic strain testing, the transducer is secured to the test system, and a specific strain is repeatedly applied and released while the electrical signal is recorded.

## 3. Results and Discussion

The performance of the sensor with three different wrinkle contacts is first investigated. The sensor sensitivity (S) can be calculated by

(1) $$S=\frac{\partial \left(\Delta R/R_{0}\right)}{\partial p}$$

where *p* is the pressure applied to the sensor, ΔR is the change in resistance when pressure is applied, and $R_{0}$ is the initial resistance at no load. The initial resistance value was measured to be approximately 200 Ω. The relative resistance changes of the three sensors at different pressures are shown in Figure 1b. Significant resistance changes are observed for the sensor with wrinkle contacts compared to the sensor without wrinkle contacts. The gradient-wrinkle sensor exhibits a wider detection range and higher sensitivity than the single-wrinkle sensor, confirming the advantages of gradient wrinkles in piezoresistive pressure sensors. The sensor with gradient wrinkles achieved the highest sensitivity value of 0.485 kPa^−1^, a result attributed to the optimization of the geometric characteristics of the gradient-wrinkle sensor, which significantly increases the contact area and provides a large number of conductive paths through the gradient-height wrinkle membrane, resulting in a sensor that exhibits excellent sensing performance.

**Figure 2:**
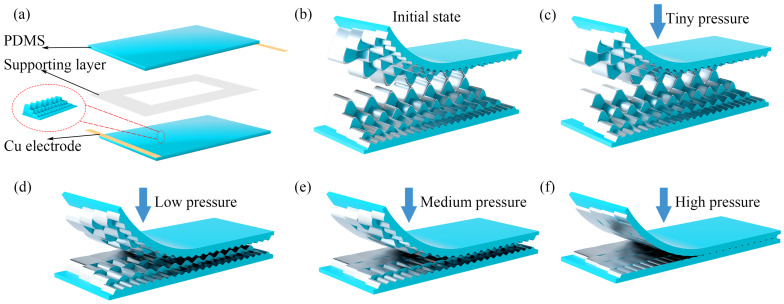
(**a**) Detailed structural diagram of the sensor. (**b**) Structural diagram of the sensor in the initial state. (**c**) Structural diagram of the sensor under tiny pressure. (**d**) Structural diagram of the sensor under low pressure. (**e**) Structural diagram of the sensor under medium pressure. (**f**) Structural diagram of the sensor under high pressure.

Meanwhile, we created a schematic diagram illustrating the interaction between the upper and lower gradient-wrinkle films under different pressures, as shown in Figure 2. In the initial state, the slight contact between the upper and lower wrinkle films results in high contact resistance. When a small amount of pressure is applied, numerous contacts occur between the AgNW-coated pleated PDMS films, causing a sharp change in the contact area and a rapid decrease in resistance. Thus, the gradient-wrinkle films achieve high sensitivity when the contact area changes significantly. At low pressure, the highest wrinkle layer is compressed first, and the resistance gradually decreases as the contact area between the wrinkle layers increases. As the pressure increases further, the contact area of the highest wrinkle layer slowly increases, new contact points appear in the medium-height wrinkle layer, and the resistance decreases further. As the load continues to increase, the contact area of the medium-height wrinkle layer increases, and new contact points begin to appear in the lower-height wrinkle layer, resulting in a further decrease in resistance. Although the highest contact point of the gradient-wrinkle structure first becomes saturated at a certain load, the new contact wrinkles can compensate for the change in the entire contact area. Therefore, a wrinkle structure with a gradient height of contact points can prolong the saturation effect in the contact area by gradually activating the contact wrinkles from the highest to the lowest layer, thereby achieving a wider sensing range.

**Figure 3:**
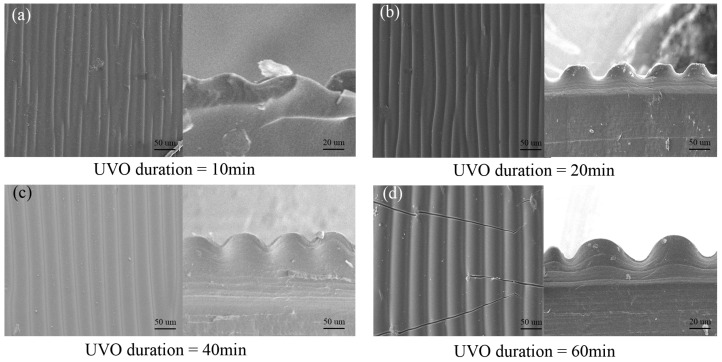
SEM images of wrinkles with different UVO treatment durations: (**a**) UVO duration of 10 min. (**b**) UVO duration of 20 min. (**c**) UVO duration of 40 min. (**d**) UVO duration of 60 min.

To further optimize the performance of the gradient-wrinkle sensor, we regulate the film morphology and the size of the wrinkle structure by controlling the UVO irradiation process. The UVO irradiation time was first controlled. The formation of wrinkles requires a composite system consisting of an elastomeric substrate and a rigid thin layer [36,37]. We uniaxially stretched four PDMS samples to the same pre-strain of 40% and then exposed them to UVO for 10, 20, 40, and 60 min, respectively. The scanning electron microscope (SEM) images in Figure 3 show the cross-sectional morphologies of the wrinkles under different UVO exposure times. It can be observed that, after 10 min of exposure, the surface wrinkles were incomplete, while, after 20 min of exposure, regular wrinkles formed on the PDMS surface, and the cross-section showed a continuous wave-like pattern. At 40 min of exposure, the PDMS surface wrinkles were more regular and pronounced. When the UVO treatment duration was increased from 20 min to 40 min, the amplitude of the wrinkle structure gradually increased, and the wavelength increased from 30 μm to 50 μm. However, when the UVO treatment duration was extended to 60 min, cracks appeared on the surface of the PDMS, which affected the performance of the piezoresistive pressure sensor.

The interaction of UVO with the PDMS leads to a change in the elasticity of the PDMS surface, transforming it from a soft elastomer to a rigid silica layer. When the UVO treatment is complete and the tensile force is removed, the lower layer of PDMS that was not exposed to the UVO returns to its original dimensions, while the rigid PDMS on the surface does not return to its original dimensions. Due to the mechanical mismatch, the PDMS surface wrinkles, and, the less elastic the surface, the more severe the mechanical mismatch, resulting in larger wrinkles.

**Figure 4:**
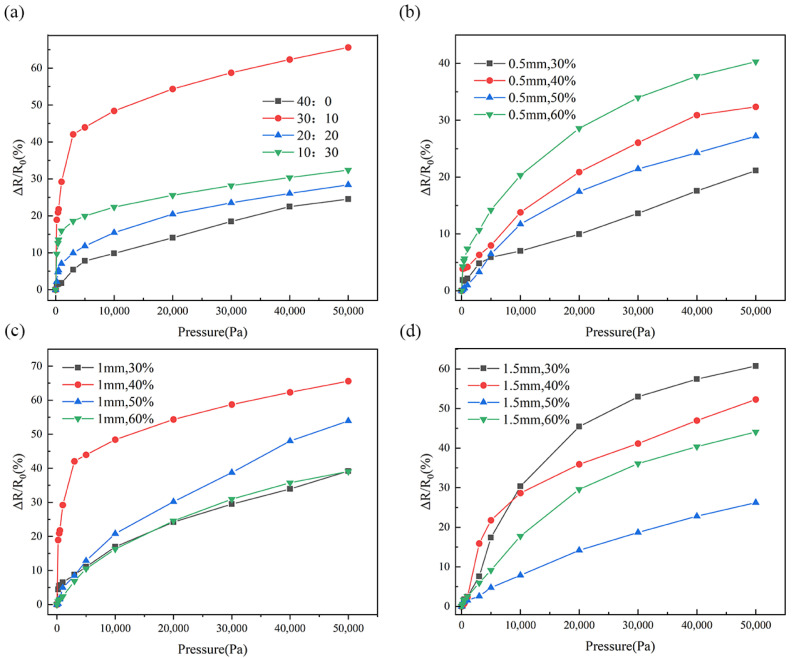
Relative resistance changes of sensors fabricated under different conditions: (**a**) resistance changes under different motor speeds. (**b**) Resistance changes under different pre-strains for the same PDMS thickness (0.5 mm). (**c**) Resistance variation with different pre-strains for the same PDMS thickness (1 mm). (**d**) Resistance variation with different pre-strains for the same PDMS thickness (0.5 mm).

The different speeds at which the stepper motor pulls the mask also affect the sensor’s performance. We fixed the UVO illumination time to 40 min and controlled the formation of different morphological gradient wrinkles by adjusting the speed at which the motor pulls the mask. Figure 4a shows the resistance changes under different pressures for sensors fabricated from different gradient-wrinkle films, with the ratios of the motor-pull mask time to the overall exposure time of the film as 40:0, 30:10, 20:20, and 10:30, all with a film thickness of 1 mm and a uniaxial pre-strain of 40%. The sensor sensitivity reaches a peak of 0.947 kPa^−1^ for a motor-pull mask exposure time of 30 min and an overall exposure time of 10 min, with a pressure range from 10 Pa to 50 kPa. A 40 min motor-pull results in incomplete wrinkle formation as certain regions of the PDMS film receive insufficient UVO exposure, preventing proper wrinkle formation in those areas. This incomplete wrinkle formation leads to a reduction in the overall deformation of the film, thereby decreasing the sensor’s sensitivity. On the other hand, an excessively short motor-pull time reduces the variation in oxidation time across different regions of the film, resulting in a smaller gradient in the wrinkle structure. Consequently, the sensor’s sensitivity is further compromised due to insufficient variation in the contact area.

Different film thicknesses and pre-strains also affect the sensitivity of the sensors. As the pre-strain increases, the amplitude of the wrinkles gradually increases, while the wavelength decreases. However, lower pre-strain results in incomplete wrinkle formation, limiting the pressure range, while higher pre-strain exceeds the critical strain for other complex modes [10]. To investigate this, we fabricated four sensors with different PDMS film thicknesses and pre-strains of 30%, 40%, 50%, and 60%, using film thicknesses of 0.5 mm, 1 mm, and 1.5 mm, a UVO processing time of 40 min, and a ratio of motor-pull mask time to overall exposure time of 30:10. The changes in relative resistance at different thicknesses and strains are shown in Figure 4b–d. When the PDMS thickness is varied, the optimum pre-strain changes accordingly. The maximum sensitivity of the sensor is achieved when the PDMS thickness is 1 mm and the pre-strain is 40%. In other configurations, the wrinkle amplitude decreases, resulting in smaller contact areas under the same pressure, thereby reducing the sensor’s sensitivity.

**Figure 5:**
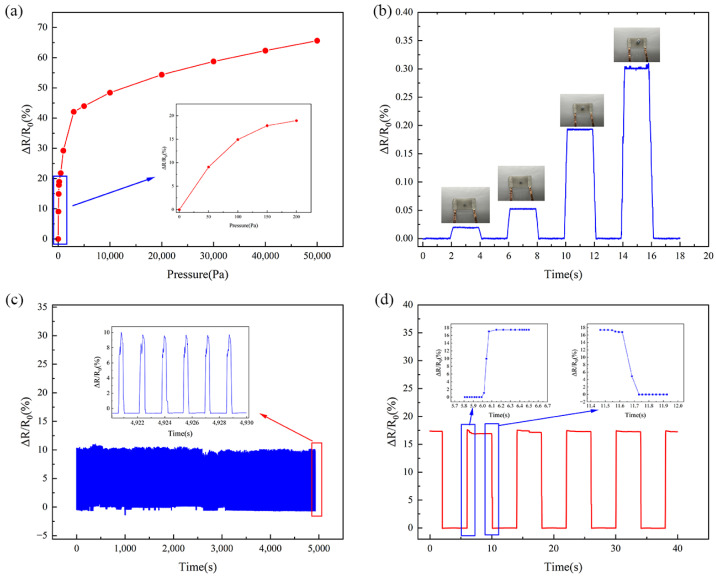
(**a**) Change in relative resistance of the sensor at different pressures. Inset: relative resistance change in the ultra-low-pressure region. (**b**) Testing the lower limit of measurement of our sensor by loading different weights. Inset: photographs of 1 g, 2 g, 5 g, and 10 g weights. (**c**) Relative resistance change of the sensor tested after 3000 loading–unloading cycles. Inset: relative resistance change for the last six cycles. (**d**) Relative resistance change of the sensor over five cycles. Inset: response time and recovery time of the sensor.

Eventually, the sensor exhibited high sensitivity of 0.947 kPa^−1^ in the low-pressure region (<200 Pa), 0.083 kPa^−1^ in the further pressure reduction region (200 Pa–3 kPa), and 0.005 kPa^−1^ in the high-pressure region (3 kPa–50 kPa^−1^), as shown in Figure 5a. To verify the sensitivity of the sensor, weights of 1 g, 2 g, 5 g, and 10 g were placed on the sensor, as shown in Figure 5b, and the accurate detection of the weight of these tiny objects proved the high sensitivity of the sensor. In order to evaluate the stability of the sensor, repeated loading–unloading cycle tests were performed, as shown in Figure 5c. The rate of change in the relative resistance of the sensor did not fluctuate significantly over 3000 cycles and remained within the same interval. The amplified portion of the last six cycles in the inset of Figure 5c showed good stability. In Figure 5d, we measured the corresponding response time of the sensor as 80 ms and the recovery time as 115 ms at a pressure of 200 Pa. Our sensor not only has a wide measurement range while maintaining high sensitivity but also features fast response and recovery times. Compared with capacitive pressure sensors with bionic petal structures [38], resistive pressure sensors with cylindrical microstructures [39], capacitive pressure sensors with hemispherical structures [40], and piezoresistive pressure sensors with hemispherical structures [41], the advantages of the gradient wave wrinkle microstructure applied by our sensor are further verified. A comparison of the performance of our sensor with other pressure sensors is shown in Table 1. Its high sensitivity and fast response make it promising for human motion monitoring and smart applications.

Despite the excellent performance of our sensor in terms of sensitivity, measurement range, and response speed, some limitations remain in practical applications. First, silver nanowires (AgNWs) may undergo shedding or aging over prolonged use, leading to a decrease in the sensor’s conductivity and stability. Second, although we have expanded the sensor’s application range by optimizing the gradient-wrinkle structure, the performance still faces limitations in ultra-high-pressure applications. When the sensor operates under extremely high pressure, the wrinkle structure may reach a point of saturation, limiting its ability to detect further pressure changes.

**Figure 6:**
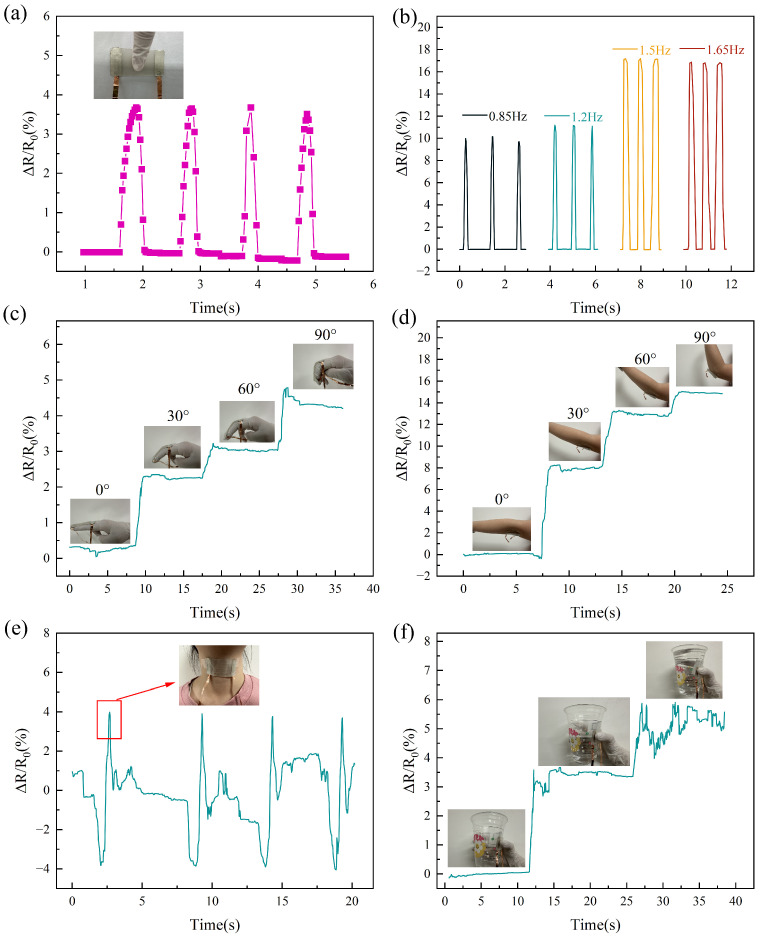
(**a**) Real-time variation in the relative resistance of the sensor during clicking behavior. Inset: photograph of the sensor during clicking behavior. (**b**) Changes in the relative resistance of the sensor when clicking at different frequencies. (**c**) Changes in the relative resistance of the sensor when the finger joint is bent at different angles. Inset: photographs of the sensor with the finger joint bent at different angles. (**d**) Changes in the relative resistance of the sensor when the elbow joint is bent at different angles. Inset: photographs of the sensor with the elbow joint bent at different angles. (**e**) Resistance change of the sensor when swallowing occurs. Inset: photograph of the sensor mounted on the human throat. (**f**) Change in relative resistance of the sensor when picking up cups of water with different weights. Inset: photographs of water cups with varying weights of water.

To verify the feasibility of the sensor in practical applications, we first placed the sensor on a flat surface, gently and quickly touched it once per second, and detected the real-time change in its relative resistance to measure the pressure from basic touch behavior, as shown in Figure 6a. The relative resistance increases rapidly when the sensor is touched and returns to its original level when the finger is released. At the same time, the sensor maintains a stable relative resistance change when subjected to dynamic stimuli at different frequencies. Figure 6b shows the frequency response of the sensor at 0.85 Hz, 1.2 Hz, 1.5 Hz, and 1.65 Hz.

With the increasing concern for personal health, the importance of real-time monitoring of human movement is becoming increasingly prominent. Flexible pressure sensors, due to their excellent performance, are capable of monitoring a wide range of body movements, which in turn aids in assessing human health in real time. Body pressure is detected by directly fixing the sensor on the skin. The sensors are fixed at the finger joints using transparent tape when the fingers are straightened, and the relative resistance of the sensors changes as the finger joints are bent at four different angles and briefly held, as shown in Figure 6c. As the bending angle of the finger joint increases, the force exerted on the sensor gradually increases, and the change in relative resistance increases accordingly. Subsequently, attaching the sensor to the elbow joint showed a change in relative resistance similar to that of the finger joint when bent at different angles, as shown in Figure 6d. In addition to behaviors such as click-touch, flexible pressure sensors have been demonstrated to detect multiple pressure levels during joint motion testing.

The sensor is also secured to the human larynx using adhesive tape, as shown in the inset of Figure 6e. As seen in the figure, when swallowing occurs, a significant change in the relative resistance of the sensor is observed, indicating that the sensor successfully detects the physiological changes associated with swallowing. Figure 6f shows the change in the relative resistance of the sensor when grasping the water cup. As shown in the inset of Figure 6f, the sensor was attached to the water cup, and pressure was applied through the hand. As the water in the cup gradually changed from empty to half-full to full, the relative resistance of the sensor changed accordingly, indicating that the sensor was able to accurately sense the change in the weight of the water cup.

This study demonstrates the broad potential of flexible pressure sensors in real-time human motion monitoring and various smart applications. The sensor’s ability to detect touch behavior, joint motion, swallowing behavior, and grasping behavior further emphasizes its effectiveness in health monitoring and intelligent robotics.

## 4. Conclusions

In conclusion, we developed a piezoresistive pressure sensor with gradient-wrinkle AgNW-coated PDMS. The sensor achieves contact through wrinkle microstructures at low pressures and exhibits excellent performance at high pressures through continuous contact area variation of the gradient-wrinkle membrane. The sensor demonstrates high sensitivity (0.947 kPa^−1^), a wide sensing range (10–50 kPa), a low detection limit (10 Pa), and excellent durability (3000 cycles). The sensor shows great potential for applications in human–computer interaction, human motion monitoring, and intelligent robotics, particularly in real-time pressure detection for touch behavior, clicking at different frequencies, joint motion, swallowing behavior, and grasping behavior recognition.

## Figures and Tables

**Table 1:** A comparative view of the performance with other flexible pressure sensors.

Material	Type	Microstructure	Sensitivity (kPa)	Response Time /Recovery Time (ms)	Cyclic Stability	Detection Limit
ZnO/PDMS [38]	Capacitive	Bionic petal	0.28	100	1000	0–10 kPa
MWCNTs-COOH/PDMS [39]	Piezoresistive	Cylindrical	1.774	-	1000	0–0.5 kPa
MWCNT/PDMS [40]	Capacitive	Hemispherical	1.99	249/83	900	0–90 kPa
SWCNT/PDMS [41]	Piezoresistive	Hemispherical	2.6	56/100	500	0–7 kPa
AgNWs/PDMS [This study]	Piezoresistive	Gradient wrinkle	0.947	80/115	3000	0–50 kPa

## Data Availability

No new data were created for this study.
